# Selective Plasmatic Amino Acid Alterations as a Potential Biomarker for Pathological Stratification in Autism Spectrum Disorders

**DOI:** 10.3390/biomedicines14010165

**Published:** 2026-01-13

**Authors:** Andrea De Giacomo, Nicoletta Lionetti, Maria Grazia Di Lago, Simonetta Simonetti, Giulia Iapadre, Alessandro Rizzello, Vittorio Sanginario, Federica Gradia, Donatella Tansella, Eustachio Vitullo, Marta Simone, Dario Sardella, Tania Lorè, Roberta Cardinali, Silvia Russo, Vincenzo Salpietro, Salvatore Scacco, Maurizio Delvecchio, Antonio Gnoni

**Affiliations:** 1 Department of Translational Biomedicine and Neuroscience, University of Bari “Aldo Moro”, 70121 Bari, Italy; andrea.degiacomo@uniba.it (A.D.G.); n.lionetti5@phd.uniba.it (N.L.); m.dilago@studenti.uniba.it (M.G.D.L.); a.rizzello20@studenti.uniba.it (A.R.); v.sanginario@studenti.uniba.it (V.S.); federicagradia@gmail.com (F.G.); d.sardella8@studenti.uniba.it (D.S.);; 2 Clinical Pathology and Neonatal Screening, Giovanni XXIII Paediatric Hospital, Giovanni XXIII University Hospital, 70126 Bari, Italy; simonetta.simonetti@policlinico.ba.it (S.S.); tania.lore@policlinico.ba.it (T.L.); roberta.cardinali@policlinico.ba.it (R.C.); silvia.russo92@gmail.com (S.R.); 3 Department of Biotechnological and Applied Clinical Sciences, University of L’Aquila, 67100 L’Aquila, Italy; giuliah@live.it (G.I.); vincenzo.salpietrodamiano@univaq.it (V.S.); maurizio.delvecchio1@univaq.it (M.D.); 4 Clinical Pathology and Microbiology Unit, Madonna delle Grazie Hospital, 75100 Matera, Italy; evitullo@asmbasilicata.it; 5 Department of Precision and Regenerative Medicine and Jonica Area (DiMePre-J), University of Bari “Aldo Moro”, 70121 Bari, Italy; marta.simone@uniba.it

**Keywords:** autism spectrum disorder (ASD), amino acid biomarkers, metabolomic analysis, phosphoethanolamine, glutamate, aspartate, severity stratification

## Abstract

**Background**: Autism Spectrum Disorders (ASD) are neurodevelopmental disorders characterized by repetitive behaviors and social interaction deficits. While the severity of ASD is classified into levels (1–3) by the DSM-5, reliable circulating biomarkers to differentiate these levels are lacking. This retrospective pilot study examines plasma amino acid levels in children with ASD to identify the potential biomarkers of disease severity. **Methods**: Plasma samples from 30 children diagnosed with ASD (24 males, 6 females, aged 3–12 years) were analyzed. Participants were stratified into two groups based on the Autism Diagnostic Observation Schedule Calibrated Severity Score (ADOS CSS): Group 1, presenting with mild symptoms (Level 1, *n* = 11), and Group 2, characterized by moderate-to-severe symptoms (Levels 2–3, *n* = 19). This was further confirmed by the identification of electroencephalogram (EEG) anomalies (21.1%) and magnetic resonance imaging (MRI) abnormalities (5.3%), which were detected exclusively in Group 2 and absent in Group 1. Amino acid levels were measured by ion-exchange chromatography. Statistical analyses (Mann–Whitney U test and chi-square test) were used to compare AA levels between groups. **Results**: Statistically significant differences were observed in the levels of phosphoethanolamine, aspartic acid, and glutamic acid between the two groups. These amino acids (AA) were significantly higher in the moderate-to-severe symptoms group (Levels 2–3) compared to the mild symptoms group (Level 1) (*p* < 0.05). All AA values remained within age-appropriate reference ranges. **Conclusions**: Plasma levels of phosphoethanolamine, aspartic acid, and glutamic acid may serve as potential biomarkers for ASD severity in children. Results from this exploratory analysis suggest that AA profiling could differentiate ASD severity and identify specific metabolic pathways, such as excitatory neurotransmission and phospholipid turnover. Further studies with larger cohorts are necessary to validate these findings and explore the role of AAs in ASD pathophysiology.

## 1. Introduction

Autism Spectrum Disorders (ASD) are a group of neurodevelopmental disorders characterized by restricted, repetitive behavioral patterns and deficits in communication and social interaction. According to recent updates, the prevalence of ASD stands at approximately 1% globally in the pediatric age group, with a male-to-female ratio of 4.2:1 [1].

ASD classification encompassed five Pervasive Developmental Disorders: Autism, Asperger’s Syndrome, Pervasive Developmental Disorders—Not Otherwise Specified, Rett Disorders, and Child Disintegrative Disorder [2]. However, due to the considerable variability in their symptoms, they are now consolidated under the broader category of ASD [3]. Currently, each individual diagnosed with an ASD is further classified into three different subgroups of severity based on their clinical presentation and the extent of support required in daily activities [4]. The subgroups of ASD range from level 1, which include individuals with milder symptoms, to level 3, representing those with the most severe ASD presentations, as defined by the DSM-V [2]. The underlying etiology of ASD remains unclear, as various factors are recognized spanning from genetic, to hormonal, prenatal, and perinatal mechanisms [5,6,7]. Concerning genetic factors, such as copy number variations in different chromosomal loci such as 15q11-q13, 16p11.2, and 22q11.21, have been linked to ASD [8,9]. Among the non-genetic factors implicated in the pathophysiology of ASD, an early imbalance between excitation and inhibition (E/I) at the cortical level has been speculated before. A significant trend towards increased excitation in circuits involved in specific cognitive functions (e.g., language and sensory processing) has been identified in people living with ASD [10,11,12].

ASD diagnosis relies primarily on two key components: (i) a detailed developmental milestones history provided by the parents/caregivers, and (ii) observation and/or evaluation of the individual’s interactions with relatives and other individuals [13]. Diagnostic interviews and observational assessments are integral to this process [14,15]. To date, no circulating diagnostic biomarkers are widely available for ASD. Several studies have attempted to fill this gap investigating metabolic profiles in various biological matrices, such as blood and urine, of individuals affected with ASD compared to healthy controls [16,17,18,19,20]. These papers have reported differences in metabolite levels between ASD individuals and healthy controls (e.g., glutamate, tryptophan, carnosine, and carnitine) [20,21,22,23,24,25,26,27,28]. Although these studies may offer insights into potential contributions or cofactors in the development of ASD, it should be reported that conflicting results have been oserved [16,17,18,19,20].

Currently, there is a lack of studies investigating circulating metabolite levels in individuals affected with ASD, even if alterations in metabolites levels may be influenced by the severity of ASD symptoms. Here, we examined the plasma amino acid (AA) levels in two distinct groups of children diagnosed with ASD, aiming to assess whether AA levels could serve as biomarkers for disease severity.

## 2. Materials and Methods

### 2.1. Study Design

This study involved 30 children who received a formal diagnosis of idiopathic/non-syndromic ASD [29] made by qualified personnel according to the DSM-5 criteria. The sample includes 24 males and 6 females, aged between 3 and 12 years, in order to follow the male-to-female ratio of ASD. The participants were recruited from the Child Neuropsychiatry Unit of Policlinico di Bari, and all recruited children received comprehensive evaluation, which involved detailed medical history, the administration of standardized tests, and blood tests. Metabolomic analysis, specifically targeting AA, was conducted on plasma samples available as part of routine biochemical evaluations. General exclusion criteria included insufficient metabolic analysis data, absence of objective information pertaining to ASD symptoms, and the presence of genetic syndromes or chromosomal anomalies. Moreover, it should be reported that food selectivity is a frequent characteristic of autism spectrum disorder and might be considered as a possible bias when studying circulating molecule. In order to overcome this confounding factor, we assessed the adequacy of the child’s diet, based on parent report during the medical history interview, enrolling only ASD patients without food selectivity in this study.

### 2.2. Subjects

Patients were categorized into two groups based on the severity of their ASD symptoms and their corresponding disease level, as per the DSM-5 classification. The first group comprised 11 patients exhibiting mild symptoms and diagnosed with ASD Level 1 (Group 1), while the second group consisted of 19 children presenting moderate-to-severe symptoms and diagnosed with ASD Level 2 or Level 3 (Group 2). To stratify the sample, we employed the Autism Diagnostic Observation Schedule Calibrated Severity Score (ADOS CSS), with Group 1 having a mean score of 10.9 and Group 2 having a mean score of 19.5.

Group 1 comprises 10 males and 1 female, accounting for 90.9% and 9.1% of the group, respectively. The age range of this group is between 3 and 8 years, with a mean age of 3.7 ± 1.4 years (Table 1).

Group 2 consists of 14 males and 5 females, representing 73.6% and 26.3% of the group, respectively. The age range for this group is between 3 and 12 years, with a mean age of 4.3 ± 1.9 years.

It is worth noting that none of the subjects in either group received pharmacological therapy at the time of biochemical evaluation.

### 2.3. Neuropsychological Findings

Linguistic skills were evaluated using the Language assessment test. Among the participants, 2 patients (6.6%) exhibited linguistic skills impairment, both of whom were from Group 2 (10.5%). Conversely, 28 children (93.3%) showed no language impairment. Within this group, 11 patients were from Group 1 (100%) and 17 patients were from Group 2 (89.4%).

Cognitive functioning was assessed using IQ scores obtained from the non-verbal Leiter-R test, as well as IQ scores derived from the multilevel tests Wechsler Preschool and Primary Scale of Intelligence III edition and Wechsler Intelligence Scale for Children IV edition (WISC-IV). Among the participants, 14 patients (46.6%) achieved scores within the range of normality (IQ 70/75–120), with 4 out of 9 belonging to Group 1 (44.4%) and 10 out of 17 belonging to Group 2 (58.8%). Conversely, 9 patients (34.6%) exhibited intellectual disability, with 3 out of 9 from Group 1 (33.3%) and 6 out of 9 from Group 2 (35.3%). was highlighted, of which 4 out of 9 belong to group 1 (44.4%) and 10 out of 17 belong to group 2 (58.8%); in 9 patients (34.6%), an intellectual disability was highlighted, of which 3 out of 9 belong to group 1 (33.3%) and 6 out of 9 belong to group 2 (35.3%).

In total, 5 patients (16.6%) with level 3 severe, 14 patients (46.7%) with level 2 moderate, and 11 patients (36.6) with level 1 mild were identified according to DSM-5.

### 2.4. EEG and Brain MRI Findings

Within Group 2 (*n* = 19), the 21.1% present EEG anomalies without an epilepsy diagnosis, as reported in [30], while the 5.3% showed abnormal brain MRI findings. Conversely, no EEG and/or brain MRI abnormalities were detected among the 11 patients comprising Group 1.

### 2.5. Comorbidities

Only 1 out of 11 patients, belonging to Group 1 (11.1%), received a comorbid diagnosis of Specific Learning Disorder. This data may not be comparable between the two groups because the diagnosis of Specific Learning Disorder requires the exclusion of intellectual disabilities and neurosensory deficits that could account for the learning difficulties. Group 2 is characterized by a more severe clinical phenotype and presents academic difficulties attributable to impairments in cognitive and adaptive functioning rather than a Specific Learning Disorder.

### 2.6. Plasma Amino Acid Metabolomic Analysis

Blood samples were collected from patients and centrifuged (10 min at 3000 rpm) for plasma separation. All samples were collected after overnight fasting (at least 8 h).

Biological samples were deproteinized by precipitation with addition of 5% (*m*/*v*) cold sulfosalicylic acid and subsequently centrifuged at 15,000× *g* for 10 min at 4 °C. The supernatants were transferred to a 0.22 µm pore-size filter tube and centrifuged at 15,000× *g* for 10 min at 4 °C. The samples were then placed on a chilled autosampler and injected into the instrument.

The Biochrom 30+ Amino Acid Analyzer (Biochrom Ltd., Cambridge, UK) performs AA separation by ion exchange chromatography with post-column derivatization with ninhydrin and simultaneous photometric detection at two wavelengths (570 and 440 nm). AA are separated by a sequence of six lithium citrate buffers with increasing pH, ionic strength, and temperature gradient. The whole process takes about 3 h per sample. Calibration curves were prepared, and run along with each batch, with a complete standard solution (AA standards, physiological, analytical standard, acids/neutrals, basics, and L-glutamine, 2500 µM, Millipore Sigma, Burlington, MA, USA) injected at known concentrations of 250 µM, 500 µM, and 1000 µM. Plasmatic AA concentrations were expressed in µM.

### 2.7. Statistical Analysis

The results of the metabolomic analysis were compared between Group 1 and Group 2. The results are presented as median and interquartile range. Due to the non-normal distribution, data between groups were compared using the Mann–Whitney U test. Each metabolite was classified as lower, normal, or higher as compared to the normal range established by the local laboratory. The frequency of lower, normal, or higher values was compared between groups using the Chi-square test. A *p*-value < 0.05 was considered as statistically significant.

Binary logistic regression analysis was performed to assess the association between plasma amino acid concentrations and ASD severity. Severity was dichotomized as mild (Level 1) versus moderate-to-severe (Level 2–3). Each amino acid was entered independently as a continuous predictor. The results are expressed as odds ratios (ORs) with 95% confidence intervals (CIs).

## 3. Results

### 3.1. Patient Clinical Stratification

The 30 patients diagnosed with ASD were divided into two groups based on symptom severity (DSM-5) and the Autism Diagnostic Observation Schedule Calibrated Severity Score (ADOS CSS).

**Figure 1:**
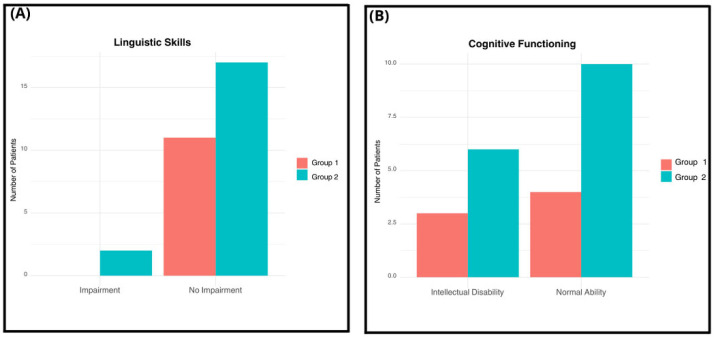
Association between severity groups and linguistic/cognitive skills. (**A**) Distribution of patients by group according to their Linguistic Skills (Impairment vs. No Impairment). (**B**) Distribution of patients by group according to their Cognitive Functioning (Intellectual Disability vs. Normal Ability). Group 1 corresponds to patients with mild symptoms (Level 1, *n* = 11); Group 2 corresponds to patients with moderate-to-severe symptoms (Levels 2–3, *n* = 19).

**Figure 2:**
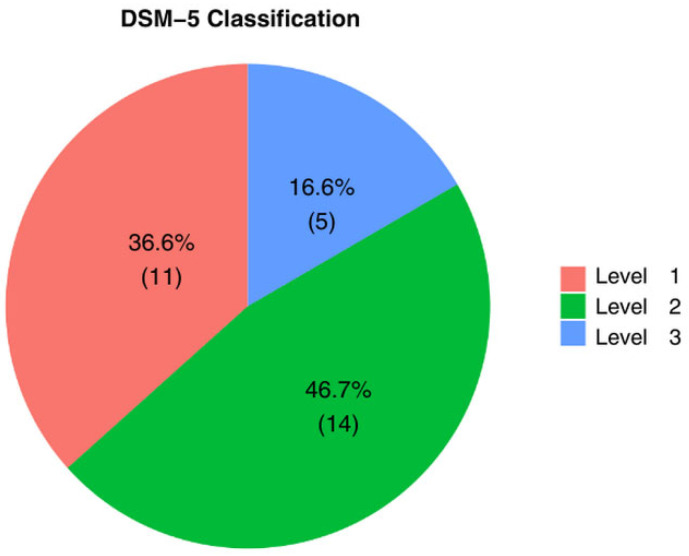
Distribution of the study cohort according to DSM-5 Classification of Severity. Pie chart illustrating the percentage and number of enrolled children (*n* = 30) diagnosed with ASD at each DSM-5 severity level: Level 1 (mild symptoms), Level 2 (moderate symptoms), and Level 3 (severe symptoms).

None of the patients in either group were receiving pharmacological therapy at the time of biochemical evaluation. Regarding comorbidities, two patients (6.6%) exhibited linguistic impairment, and both belonged to Group 2 (10.5% of Group 2). Group 1 showed no linguistic impairment (100% unimpaired) (Figure 1A). Regarding cognitive functioning, normal intellectual ability was identified in 14 patients, accounting for 46.6% of the total cohort. Descriptively, this status was recorded in 58.8% of patients in Group 2, compared to 44.4% in Group 1 (Figure 1B). Conversely, Intellectual Disability was documented in nine patients (34.6% of the total), exhibiting a similar distribution across both groups (33.3% in Group 1 and 35.3% in Group 2). The distribution of severity according to the DSM-5 was 11 patients (36.6%) Level 1, 14 patients (46.7%) Level 2, and 5 patients (16.6%) Level 3 (Figure 2).

### 3.2. Plasma Amino Acid Modifications Between Group 1 and 2

Results obtained by comparing the AA levels between Group 1 and 2 are summarized in Table 2.

**Figure 3:**
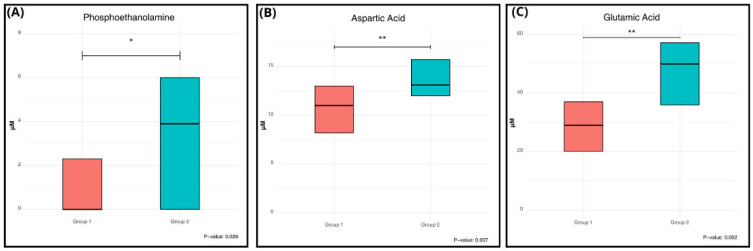
Plasmatic levels of PhEt, aspartic acid, and glutamic acid between the two severity groups. Box-plot graphs showing the concentration (µM) of specific amino acids (AAs) in Group 1 (mild symptoms, Level 1) and Group 2 (moderate-to-severe symptoms, Levels 2–3). (**A**) Comparison of PhEt levels; (**B**) comparison of aspartic acid levels; (**C**) comparison of glutamic acid levels. Asterisks indicate statistically significant differences: * *p* < 0.05; ** *p* < 0.01 (Mann–Whitney U test). The *p*-value is indicated below each plot.

Interestingly, only three AA showed a statistically significant variation: phosphoethanolamine (PhEt), aspartic acid, and glutamic acid. Figure 3 shows the significant variation, evident from the comparison of the box plots of the two groups and highlighted by *p*-values less than 0.05. (Figure 3).

These three AA were all found to be higher in Group 1 compared to Group 2, with aspartic acid showing an increment of the median value of nearly 20%, glutamic acid of 72%, and PhEt of 390%.

It should be reported that the obtained AA values reported in Table 2 were compared to the age-matched interval of the reference population, obtaining no statistical significant variations.

Binary logistic regression analysis confirmed that higher plasma concentrations of glutamic acid were significantly associated with increased odds of moderate-to-severe ASD (OR 1.084, 95% CI 1.01–1.16, *p* = 0.025). Similarly, aspartic acid (OR 1.60, 95% CI 1.09–2.36, *p* = 0.017) and PhEt (OR 1.478, 95% CI 1.02–2.14, *p* = 0.037) were positively associated with ASD severity (Table 3).

## 4. Discussion

ASD encompasses a spectrum of neurodevelopmental disorders, globally affecting approximately 1 in 88 individuals [31,32]. Increasing evidence suggests that ASD may be influenced by genetic, environmental, immunological, and biochemical factors. Among the non-genetic factors implicated in ASD pathophysiology is the proposed early imbalance between E/I at the cortical level. Currently, the latest version of the DSM-5-TR defines severity levels of ASD based on the level of support needed for the two core psychopathological domains: social communication impairments and restricted, repetitive patterns of behavior. These severity levels are categorized as Level 1 “Requiring support”, Level 2 “Requiring substantial support”, and Level 3 “Requiring very substantial support” [4]. Level 3 social communication is defined by “very limited initiation of social interactions, and minimal response to social overtures from others”. In contrast, level 1 social communication involves “difficulty initiating social interactions, and clear examples of atypical or unsuccessful response to social overtures of others.” Additionally, these severity levels are explicitly linked to varying degrees of functional impairment: Level 1 indicates that “without supports in place, deficits in social communication cause noticeable impairments”, Level 2 suggests that “social impairments are apparent even with supports in place”, and Level 3 denotes that “severe deficits in verbal and nonverbal social communication skills cause severe impairments in functioning” [4,15].

While the Severity Score has been utilized in previous studies to differentiate clinical features among individuals on the autism spectrum based on diagnostic level, developmental trajectory, and treatment response, to date, no study has explored the comparison of metabolites between these groups. This disparity in research focus could offer insights into differential treatment approaches aimed at rebalancing AA. Such insights may help in mapping the metabolic heterogeneity of ASD, directing future research towards specific biochemical targets rather than generalized treatments.

AA are organic compounds that not only constitute proteins, but play multiple functions at different biological levels (e.g., structural, cellular energetic sources, cellular differentiation, and tissue function). Dysregulation of circulating AA levels has been linked to a growing number of diseases (e.g., metabolic, cancer, cardiovascular, and neurological [33]). Emerging evidence suggests that imbalances in circulating AA constitute a significant etiological component in ASD, contributing to alterations in metabolic pathways [34,35]. A notable study investigated the plasma AA profiles of over 500 children with ASD compared to age-matched controls. While no statistically significant differences were initially observed between the two groups, heuristic algorithms revealed three distinct subsets of ASD metabotypes [36]. Furthermore, metabolic disorders, such as alterations in iron, folic acid, calcium, and vitamin D metabolism, are closely related factors that could contribute to the occurrence of ASD. These findings underscore the multifaceted nature of ASD and highlight the importance of considering metabolic factors in its etiology [37,38,39,40,41].

In this study, circulating AA quantification analysis was employed due to their relevance in elucidating the pathophysiology of ASD. This study marks the first comparison of plasmatic AA profiles in very young children with ASD across different severity levels.

The gold standard tools for diagnosis include the Autistic Diagnostic Interview-Revised (ADI-R) and the Autistic Diagnostic Observation Schedule-2 (ADOS) [42]. Our findings reveal a statistically significant increase in three AA within the most severe group compared to the mild group. Specifically, the median levels of PhEt increased by 390%, glutamic acid by 72%, and aspartic acid by 19% in the most severe group compared to the mild group. PhEt is a phosphomonoester metabolite implicated in the biosynthesis processes of phospholipids and is produced by their breakdown. PhEt is involved in several key biological processes, including the stabilization of cellular membranes, regulation of membrane trafficking, and intracellular signaling. Its presence is particularly important in the cellular membranes of the nervous system, where it contributes to multiple functions like synapse formation and neuronal function [43,44]. In a previous work, a similar increment of circulating PhEt levels was found in ASD [45]. However, it should be pointed out that in this study the authors compared the ASD plasma with age matched controls. Moreover, the mean age of ASD patients investigated by Bala et al. was the double compared to our patients (8.38 vs. 4.00 years) and also the age range was broader (2–18 vs. 3–12 years) [40]. The reported PhEt increase in most severe ASD group is not uniquely determined, as PhEt is both a synthesis product, but also a reagent in several biochemical reactions. One of the PhEt synthesis routes starts from sphinganine 1-phosphate and is catalyzed by sphinganine-1-phosphate aldolase; another involves the ethanolamine kinase, which catalyzed the reaction between ATP and Ethanolamine [43,44]. However, PhEt is itself a precursor to numerous macromolecules, in particular phosphatidylethanolamine is an intermediate in the phospholipid synthesis pathway known as PE. This molecule plays various critical roles within human cells, acting: (i) into the composition of cell membranes thereby influencing their structure and fluidity; (ii) in blood coagulation, aiding in platelet formation and the coagulation cascade.

Moreover, PE is implicated in autophagy, a cellular process responsible for the degradation and recycling of cellular components. It also contributes to cell signaling pathways and processes associated with apoptosis, or programmed cell death [43,44].

Notably, in a recent paper it has been reported that the plasmatic phospholipase A2 and PE ratio was increased in a cohort of ASD patients aged like ours, compared to age-matched controls, thus proposing this ratio as a promising ASD biomarker [46].

Recent studies have focused on hypothesizing a role for the intestinal microbiome and its influence on the gut–brain axis in children diagnosed with ASD. The underlying concept is the neuroinflammation theory, which posits that neurodevelopmental disorders share an inflammatory substrate that may result from various conditions, including intestinal dysbiosis or altered composition of the intestinal microbiome [47,48]. It should be remembered that approximately 90% of neurotransmitters, such as serotonin, are produced in the gut [49]. Although indirectly, the metabolism of PhEt can influence the production of short-chain fatty acids by gut microbiota, altering the quantity and composition of the final biometabolites that will cross the blood–brain barrier [50]. In turn, the metabolism of phospholipids containing PhEt can lead to the release of fatty acids, which may play significant roles in energy metabolism and the maintenance of oxidative balance [51]. Notably, in the light of a nutraceutical strategy on ASD, in a preclinical model, the authors demonstrated that caffeine administration significantly lowered the PhEt levels; this was mediated through a mitochondrial-mediated mechanism related, among other factors, to fat storage [52]. In the light of the interconnection between PhEt and PE a wide plasmatic metabolomic survey on caffeinated and decaffeinated consumers, indicated a significant decrement of PE levels in the caffeine group [53].

Herein, we observed a significant elevation in circulating glutamate levels in Group 2 compared to Group 1. Glutamate, whose metabolism is linked to that of aspartate, serves as the primary excitatory neurotransmitter in the brain; thus, playing a pivotal role in the central nervous system (CNS) development and function. Glutamate, a non-essential AA, facilitates enhanced communication between neurons, contributing to both learning and memory processes [54]. Dysregulation of the glutamatergic system is known to play a key role in ASD, and various studies have demonstrated that the underlying pathophysiology of ASD often involves an imbalance in excitatory and inhibitory neurotransmitters [55]. Interestingly, altered levels of glutamate concentration (i.e. elevation and/or depression) in the CNS have been reported in pediatric and adolescent individuals diagnosed with ASD compared to controls [56,57]. To date, conflicting results have been reported regarding circulating glutamate levels in children with ASD compared to age-matched healthy controls [34,35,58,59].

NMDA receptors are highly represented in the fronto-striatal circuit, which is hyperexcitable in individuals with autism spectrum disorder, especially in those who exhibit typically complex stereotypies and pervasive, restricted and repetitive behaviors [60].

The severity of motor stereotypies is correlated with the disorder’s severity and the level of support required, coded as the diagnosis level. In this case, our findings are consistent with the current literature, highlighting an increase in glutamate in Group 2. In future studies we plan to use standardized scales to characterize the sample’s stereotypies and correlate them with the amino acid metabolomic profile [61].

There is also growing evidence that the fronto-striatal circuit plays a role in the processing of social reward. Glutamatergic projections from the medial prefrontal cortex to the nucleus accumbens, a key region of the ventral striatum, are activated during social preference tasks and can drive social approach behavior when optogenetically stimulated, suggesting a causal role for this pathway in encoding the motivational value of social stimuli [62].

Furthermore, Aspartate was found to be elevated in the plasma of patients belonging to Group 2. Although Aspartate is a non-essential AA, it cannot traverse the blood–brain barrier, so it must be synthesized in situ. Aspartate participates in a plethora of metabolic pathways, including the urea cycle and the citric acid cycle, which are vital for energy homeostasis [63]. Additionally, aspartate functions as a buffer in maintaining the body’s acid–base equilibrium, also regulating blood pH levels. Consistent with the observations for glutamate, circulating plasma concentrations of aspartate in the present study demonstrated a downward trend [64,65,66,67] in children with ASD relative to age-matched controls.

It should be remarked that the AA levels of Group 1 and Group 2 were within the reference interval of healthy age-matched, even for those AAs that were statistically different between the two groups, thus indicating that the enrolled patients did not have AA disbalance (e.g., Inborn Errors of Metabolism). Consequently, the association between symptom severity and variations within this physiological range suggests a functional neuromodulatory mechanism rather than a frank pathological excess. This implies that even subclinical elevations, even within normal range, in excitatory amino acids or membrane precursors may impact the clinical phenotype by perturbing the homeostatic regulation of neural circuits in susceptible individuals.

This study has limitations and caveats. Firstly, the sample size was limited, as we studied the AA profiles of 30 children. However, this could be regarded as a pilot study to generate valuable insights for further validation in larger clinical studies. Additionally, we excluded patients with ASD due to other (secondary) causes, which may limit the generalizability of our findings. While we acknowledge the above points and limitations, it is worth noting that there are currently no investigations on this specific topic, making our pilot study an important first step in providing exploratory observations and findings within this field of research.

## 5. Conclusions

Our study offers novel insights into the ASD research field, indicating that the plasma levels of PhEt, aspartic acid, and glutamic acid may serve as promising candidate biomarkers for severity stratification in ASD. Moreover, these three AAs highlight critical metabolic nodes, specifically involving excitatory neurotransmission and membrane lipid metabolism, that warrant further exploration to understand the biological mechanisms of severe ASD. Further testing in larger cohorts is necessary to validate their utility for diagnosis and to understand their role in the disorder’s pathophysiology.

## Figures and Tables

**Table 1:** Demographic and clinical characteristics of study participants. Demographic and clinical characteristics of the two groups of participants, categorized by ASD symptom severity and DSM-5 classification. Group 1 comprises children with mild ASD symptoms (Level 1), while Group 2 includes children with moderate-to-severe symptoms (Levels 2/3). The table presents the number of patients, symptom severity, DSM-5 classification, mean Autism Diagnostic Observation Schedule calibrated severity score (ADOS CSS), gender distribution, age range, mean age with standard deviation (SD), and pharmacological therapy status for each group.

	Group 1 (ASD Level 1)	Group 2 (ASD Level 2/3)
Number of patients (*n*)	11	19
Symptom severity	Mild	Moderate-severe
DSM-5 classification	Level 1	Level 2/3
Mean ADOS CSS score	10.9	19.5
Gender distribution (Males)	10 (90.9%)	14 (73.6%)
Gender distribution (Females)	1 (9.1%)	5 (26.3%)
Age range (years)	3–8	3–12
Mean age ± SD (years)	3.7 ± 1.4	4.3 ± 1.9
Pharmacological therapy	None	None

**Table 2:** Results of blood assay. Data are expressed as µmol/L and reported as median (interquartile range); sample size were reported for each parameter. Statistically different values are reported in bold, and median percentage increase, relative to group 2 as 100%, is shown in square brackets.

METABOLITE	GROUP 1 (11 Patients)	GROUP 2 (19 Patients)	*p*
**Aspartic Acid**	**11.0 (8.2/13.0)**	**13.1 (12.0/15.7) [19.1%]**	**0.007**
**Glutamic Acid**	**29.0 (20.1/37.0)**	**50.0 (36.0/57.2) [72.4%]**	**0.002**
**Phosphoethanolamine**	**0.0 (0.0/2.3)**	**3.9 (0.0/6.0) [390%]**	**0.026**
1-Metyl-Histidine	8.8 (3.6/13.5)	8.7 (4.1/16.1)	0.832
3-Metyl-Histidine	5.8 (1.5/10.4)	3.0 (2.0/9.6)	0.371
Alanine	325.8 (258.1/381.8)	315.5 (248.3/371.0)	0.8
Anserine	0.0 (0.0/0.0)	0.0 (0.0/0.0)	0.832
Arginine	37.6 (33.8/66.0)	54.0 (43.0/62.5)	0.112
Asparagine	78.4 (64.7/93.0)	77.5 (66.0/94.3)	0.767
Carnosine	0.0 (0.0/0.0)	0.0 (0.0/0.0)	0.395
Citrulline	39.6 (25.8/41.5)	38.7 (33.7/44.6)	0.641
Cystine	42.8 (34.0/45.4)	39.5 (34.7/42.1)	0.641
DL-Allohydroxylysine	0.0 (0.0/0.0)	0.0 (0.0/1.0)	0.158
DL-Cystathionine	0.0 (0.0/2.0)	0.0 (0.0/0.0)	0.641
GABA	0.0 (0.0/0.0)	0.0 (0.0/0.0)	0.703
Glutamine	520.3 (475.2/562.0)	543.6 (509.5/575.8)	0.35
Glycine	233.9 (218.7/276.9)	236.2 (190.3/272.7)	0.641
Histidine	88.0 (78.3/92.9)	86.3 (83.0/95.2)	0.672
Homocysteine	0.0 (0.0/0.0)	0.0 (0.0/0.0)	0.832
Hydroxyproline	22.0 (11.0/25.0)	16.0 (12.0/19.0)	0.268
Isoleucine	66.3 (60.4/92.0)	78.4 (71.1/89.1)	0.158
Leucine	134.1 (109.1/183.9)	151.7 (138.4/171.8)	0.268
Lysine	154.0 (145.8/191.0)	165.7 (152.0/191.1)	0.372
Methionine	25.2 (21.1/30.3)	27.0 (22.9/32.0)	0.445
Ornithine	82.8 (73.4/113.0)	101.0 (79.1/118.2)	0.185
Phenylalanine	64.4 (52.9/69.0)	66.7 (55.7/74.8)	0.232
Phosphoserine	9.8 (0.0/19.0)	11.8 (4.9/17.0)	0.661
Proline	156.0 (138.0/173.0)	165.0 (137.0/200.0)	0.641
Serine	124.8 (95.5/149.5)	146.8 (124.7/165.7)	0.112
Taurine	97.0 (92.5/156.9)	129.4 (105.3/141.9)	0.471
Threonine	116.1 (103.1/141.1)	130.7 (119.5/141.3)	0.328
Tryptophan	45.4 (39.0/69.0)	52.0 (42.0/68.2)	0.735
Tyrosine	71.4 (58.6/82.4)	76.8 (64.9/88.6)	0.307
Valine	220.6 (188.5/277.4)	244.3 (215.1/275.2)	0.328
α Amino-adipic Acid	0.0 (0.0/2.3)	0.0 (0.0/0.6)	0.866
α Amino-butyric Acid	20.5 (13.8/25.0)	24.4 (16.0/29.8)	0.218
β-Alanine	0.0 (0.0/0.0)	0.0 (0.0/5.9)	0.395

**Table 3:** Binary logistic regression models were performed separately for each plasmatic amino acid. Amino acid concentrations were entered as continuous variables. Odds ratios (ORs) represent the increase in odds of moderate-to-severe ASD (Level 2–3) per unit increase in plasma amino acid concentration. Outcome: ASD severity (0 = Level 1, mild; 1 = Level 2–3, moderate–severe).

METABOLITE	Odds Ratio (OR)	95% CI	*p*-Value
Glutamic Acid	1.084	1.01–1.16	0.025
Aspartic Acid	1.600	1.09–2.36	0.017
Phosphoethanolamine	1.478	1.02–2.14	0.037

## Data Availability

No datasets were generated or analyzed during the current study.

## Abbreviations

The following abbreviations are used in this manuscript:

AA	Amino acids
ADI-R	Autistic Diagnostic Interview-Revised
ADOS	Autism Diagnostic Observation
ADOS-2	Autistic Diagnostic Observation Schedule-2
ASD	Autism Spectrum Disorder
CNS	central nervous system
CSS	Schedule calibrated severity score
DSM-5	Diagnostic and Statistical Manual of Mental Disorders (5th ed.)
DSM-5 TR	Diagnostic and Statistical Manual of Mental Disorders, Fifth Edition, Text Revision
EEG	Electroencephalogram
E/I	excitation and inhibition
IQ	Intelligence quotient
Leiter-R	Leiter International Performance Scale—Revised
MRI	Magnetic resonance imaging
PE	phosphatidylethanolamine
PhEt	Phosphoethanolamine
WISC-IV	Wechsler Intelligence Scale for Children—4th Edition
